# Effect of Cocaine on Potassium-Evoked Release of Glutamate From Fetal Rat Brain Synaptosomes

**DOI:** 10.7759/cureus.30075

**Published:** 2022-10-08

**Authors:** Donald H Penning, Brian Jones, Mohamed Fayed, Xiaoxia Han, Chaya Brodie

**Affiliations:** 1 Anesthesiology, Henry Ford Health System, Detroit, USA; 2 Anesthesiology and Perioperative Medicine, Private Practice, Eugene, USA; 3 Anesthesiology, Pain Management and Perioperative Medicine, Henry Ford Health System, Detroit, USA; 4 Research, Henry Ford Health System, Detroit, USA; 5 Neurobiology, Bar-Ilan University, Ramat Gan, ISR

**Keywords:** fetal death, maternal drug abuse, fetal growth restriction, rat study, congenital abnormalities, placenta diseases, brain hypoxia, brain development, glutamate excitotoxicity, cocaine

## Abstract

Introduction

Cocaine use during pregnancy can affect fetal brain development. A fetal brain injury could happen from the direct effect of cocaine on the developing brain or from the reduction of placental perfusion from vasoconstriction, which may lead to hypoxia-ischemia. A potential mechanism for brain injury could be due to a neurotransmitter imbalance within the brain, especially glutamate. In an immature rat brain synaptosome model, we explored the additive effect of cocaine alone on glutamate release and the effect of cocaine combined with simulated hypoxic depolarization using potassium as a surrogate.

Method

Rat pups' brains were dissected and placed on a chilled petri dish. They then entered the experimental protocol. The suspended synaptosomes were divided equally into four experimental groups (control, high potassium "surrogate to hypoxic stimulation," cocaine, and cocaine + high K). Reversed-phase high-performance liquid chromatography analyzed glutamate with fluorescent detection

Results

The glutamate level was lowest in the cocaine-only group, with a level of 1.96 × 10^4^, compared to the control and high potassium group. However, combining cocaine with high potassium seemed to generate a synergistic effect, achieving the highest glutamate level of all groups with a value of 5.31 × 10^4^.

Post hoc Conover's test for multiple pairwise-comparison between groups was done. In comparing various solutions to control, we did not find a statistically significant difference with the cocaine-only solution with a p-value of 0.074. Also, on comparing various other solutions to each other, there was no statistically significant difference between cocaine vs. cocaine + high potassium a p-value of 0.074.

Conclusion

Our data support the conclusion that cocaine alone does not induce glutamate release from fetal rat brain synaptosomes. Exposure to high potassium does lead to glutamate release. However, cocaine greatly enhances glutamate release in the presence of high potassium levels. This could explain how cocaine affects brain maturation during pregnancy with a low oxygen tension environment in the placenta. This hypothesis should be tested in vivo.

## Introduction

The reported incidence of fetal cocaine exposure in the United States varies widely, but an estimate from 2009 is that 7.5 million children in the United States were exposed prenatally to cocaine [1]. The use of cocaine can result in spontaneous abortions, placental abruption, stillbirths, and premature delivery [2]. Reduction of placental perfusion from vasoconstriction may lead to hypoxia-ischemia in the fetus. Even transient episodes of fetal hypoxemia have been shown to disrupt the maturation of pyramidal neurons in the ovine hippocampus [3]. Cocaine may also directly affect the developing brain, resulting in postnatal neurologic dysfunction and abnormal behavior [4]. One such potential mechanism for brain injury may result from an alteration in the brain's function or quantity of neurotransmitters after cocaine exposure. 

Previous work has shown that L-glutamate, the major excitatory neurotransmitter in the adult brain, plays a crucial role in brain development. As an optimal level of glutamate is required for normal neuronal development, fetal cocaine exposure may alter necessary homeostatic concentrations or functions, resulting in the abnormal neurological development of the fetal brain. Previous investigations by the author have shown that bolus maternal cocaine administration does not significantly increase fetal sheep cortical glutamate concentration [5]. Several studies measured the effect of cocaine on glutamate receptors. One study showed cocaine attenuated the sodium current but did not affect potassium and calcium currents [6]. The N-methyl-D-aspartate receptor-mediated excitatory postsynaptic currents were reduced by neuropeptide galanin but not cocaine [7]. Another study showed that repeated cocaine exposure produces an enduring reduction in mGluR2/3 function that regulates glutamate release in the nucleus accumbens [8]. It may be that simultaneous insults, such as other drugs or mild hypoxia/ischemia, can be additive to the harmful effects of cocaine leading to injury. 

These simultaneous insults are more than theoretical since cocaine abusers often use other drugs, including cigarettes and ethanol [9]. These factors have been implicated in impaired placental gas exchange or toxicity in their own right, for example, fetal alcohol syndrome [10]. We explored the additive effects on glutamate release of cocaine plus simulated hypoxic-induced depolarization using potassium in an immature brain synaptosome model.

## Materials and methods

Animal housing and surgery

All experiments were conducted according to the National Institutes of Health Guide for the Care and Use of Laboratory Animals. The study was approved by the Duke Animal Care Committee. Pregnant Wistar rats (Harlan, Raleigh, North Carolina), weighing between 250 and 300 g, were individually housed and maintained on a 12:12-h light/dark cycle (7:00 AM/7:00 PM) with free access to food and water. All experimentation was conducted during the light period.

Test solutions

Various solutions were prepared as follows: (1) Artificial cerebrospinal fluid (aCSF) was prepared using Nanopure® (Nanopuretech, South Carolina, USA) water, buffered to pH 7.4 according to the following recipe: sodium chloride 118 mM; potassium chloride 4.8 mM; magnesium sulfate 1.2 mM; potassium phosphate 1.2 mM; calcium chloride 2.5 mM; sodium bicarbonate 25mM; glucose 11 mM. (2) Cocaine was prepared in aCSF at 1mg/ml concentration. (3) High potassium was prepared by substituting potassium chloride 35 mM and sodium chloride 88 mM.

Synaptosomes isolation

Synaptosomes were prepared by a protocol described by Breukel et al. [4]. Briefly, pregnant Wistar rats (n=10) were killed by decapitation, and the rat pups (6 pups per litter) were rapidly removed, then their brains were dissected and placed on a chilled petri dish. The cortex was then dissected and placed in a cold 0.32 M sucrose homogenization buffer (sucrose 0.32M, Hepes 5M, and ethylenediaminetetraacetic acid (EDTA) 0.1M, pH 7.5). Each experiment was performed on the pooled brains from one litter. 

Preparation and grouping of the samples

**Figure 1:**
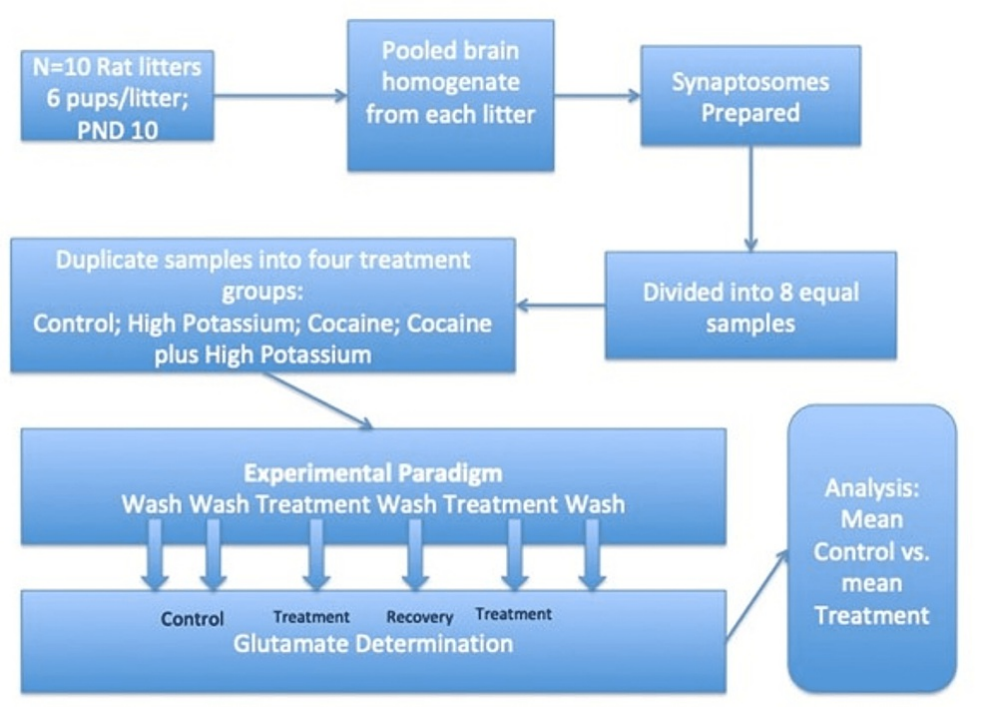
Study technique.

The tissues were then homogenized gently and slowly in cold 0.32 buffer, 80% w/v at about 400 rpm in a glass/Teflon homogenizer 8-10 times. The homogenate was centrifuged for 10 minutes at 1000g at four degrees Celsius. The supernatant was then removed and centrifuged at 17,000 g for 20 minutes at four degrees Celsius. The supernatant was discarded, and the synaptosome pellet was suspended in the original volume of sucrose (3ml). The synaptosomes were washed and spun down several times to achieve a stable baseline glutamate release. They then entered the experimental protocol (Figure 1): The suspended synaptosomes were divided equally into eight test tubes. There were four experimental groups run in duplicate. 

The four experimental groups were as follows: (1) control, (2) high potassium (high-K; 35 mM potassium was determined to produce a maximal response in pilot experiments), (3) cocaine, and (4) cocaine + high K. Experiments were carried out at 37 degrees. The divided synaptosomes were suspended in aCSF to incubate for 10 minutes and gently centrifuged. This was repeated three times. The fourth re-suspension was with the experimental solution. After another 10-minute incubation period, the aCSF was decanted, and glutamate levels were determined (see Figure 1). 

Quantification of glutamate

The glutamate concentration in the samples was determined using high-performance liquid chromatography (HPCL) with fluorometric detection. The samples were collected into 10 μl of 0.05 M hydrochloric acid (HCl) containing two pmol of homoserine as an internal standard. A reversed-phase column (10 cm, three μm ozone depleting substance (ODS); Bioanalytical Systems, Inc., West Lafayette, Indiana, United States) was used to separate the amino acids, and pre-column derivatization of amino acids with phthalaldehyde was performed using a model 540 autosampler (ESA laboratories, Chelmsford, Massachusetts, United States). Glutamate was detected by a fluorescence spectrophotometer using an excitation wavelength of 336 nm and an emission wavelength of 420 nm. Glutamate was expressed as peak height. Previous work gave a lower limit of sensitivity of 9 pmol.

Statistical analysis

The Friedman and post hoc Conover tests were used to determine whether glutamate levels differed among the four groups [11,12]. Data are present as mean ± standard error of the mean (SEM). P values less than 0.05 (two-tailed) were regarded as statistically significant. All statistical analysis was performed using R software version 3.5.2 [13].

## Results

Table 1 shows summary statistics for the relative glutamate levels of each experimental group. We can see that the glutamate level was lowest in the cocaine-only group with a level of 1.96 × 10^4^, compared to the control and high potassium group. However, combining cocaine with high potassium, seemed to generate a synergistic effect as this achieved the highest glutamate level of all groups with a value of 5.31 × 10^4^ (see Table 1).

**Table 1:** Summary statistics for the glutamate levels of each experimental group. SEM: standard error of the mean, CI: confidence interval

	Control	High Potassium	Cocaine	Cocaine + High Potassium
Mean glutamate concentration	2.51 × 10^4^	4.03 × 10^4^	1.96 × 10^4^	5.31 × 10^4^
Standard deviation	1.99 × 10^4^	1.95 × 10^4^	1.43 × 10^4^	3.12 × 10^4^
SEM	0.55 × 10^4^	0.54 × 10^4^	0.39 × 10^4^	0.86 × 10^4^
Lower 95% CI of mean	1.30 × 10^4^	2.85 × 10^4^	1.10 × 10^4^	3.42 × 10^4^
Upper 95% CI of mean	3.71 × 10^4^	5.21 × 10^4^	2.83 × 10^4^	7.20× 10^4^

As the Friedman test was significant (p-value <0.001), we performed post hoc Conover’s test for multiple pairwise-comparison between groups. In comparing various solutions to control, we didn’t find a statistically significant difference with a cocaine-only solution with a p-value of 0.074. Also, on comparing various other solutions to each other, there was no statistically significant difference between cocaine vs. cocaine + high potassium (see Table 2).

**Table 2:** Conover’s test of multiple comparisons following a significant Friedman test.

Comparison	Adjusted p-value (Dunn’s test)
Comparing to control:	
High Potassium vs. control	<0.001
Cocaine vs. control	0.074
Cocaine + High Potassium vs. control	<0.001
Comparing other various solutions:	
Cocaine vs. High Potassium	<0.001
Cocaine vs. Cocaine + High Potassium	<0.001
Cocaine + High Potassium vs. High Potassium	0.074

**Figure 2:**
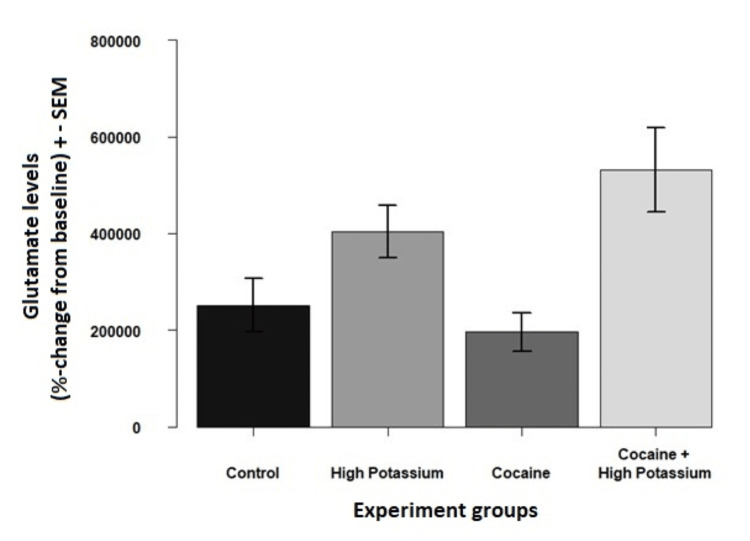
Mean glutamate levels ± standard error of the mean. SEM: standard error of the mean

Figure 2 demonstrates a graphic representation of glutamate levels among various test solutions. The graph shows that control and cocaine solutions confidence intervals cross each other, indicating no statistically significant difference between both samples. This confidence interval crossing is not observed in other testing solutions (see Figure 2). 

## Discussion

As expected, high-potassium exposure evoked significant glutamate release from our synaptosome preparation. Cocaine has been shown to suppress glutamate release in previous reports [7,8]. However, surprisingly the combination of cocaine and high potassium resulted in a significant glutamate efflux, over and above potassium-stimulated release alone.

We studied the effect of cocaine on the developing brain because of the concern over the detrimental effects of cocaine on brain maturation in fetuses. Glutamate release from hypoxia/ischemia has been implicated in apoptotic and non-apoptotic neuronal injury [14]. Our model of potassium-evoked release simulated the effect of hypoxia/ischemia in the brain [5]. Our results suggest that while cocaine had minimal effects on the resting brain, it potentiated the effects of simulated hypoxia/ischemia. This finding may partially explain the disparate clinical reports on the impact of cocaine in intact animals and humans. Available evidence suggests that cocaine induces cerebral vasoconstriction and cerebral ischemia [15]. However, our results suggest a mechanism whereby even a moderate degree of hypoxia/ischemia, which might not reach clinical significance alone, could prime the brain towards injury in the presence of cocaine. This clinical scenario could even be more evident in the pregnant uterus, where the placenta already has low oxygen tension and behaves in an environment like ischemia [16]. This could partially explain the hazardous effects of cocaine on fetuses. 

These findings need to be confirmed in more complex systems. Glia, especially astrocytes, play an essential role in glutamate reuptake, and it is unclear if their presence would mitigate the accumulation, and hence the effect, of glutamate in the brain.

## Conclusions

Our data support the conclusion that cocaine alone does not induce glutamate release from fetal rat brain synaptosomes. Exposure to high potassium, a surrogate of hypoxic stimulation, does lead to glutamate release. Cocaine greatly enhances this effect. A low oxygen environment exists in normal pregnancy, especially in the placenta. Numerous medical conditions can worsen this situation, and the presence of cocaine could lead to toxic levels of glutamate. This hypothesis should be tested in vivo.
